# Anxiety, depression, and influencing factors of STD clinic clients in Shanghai: a cross-sectional study

**DOI:** 10.3389/fpubh.2026.1775944

**Published:** 2026-06-24

**Authors:** Yanting Wang, Peng Hu, Zhenyu Wang, Junyi Huang, Limeng Yan

**Affiliations:** 1 Shanghai Skin Disease Hospital, School of Medicine, Tongji University, Shanghai, China; 2 Center for Disease Control and Prevention (Health and Sanitation Supervision Institute), Changning District, Shanghai, China

**Keywords:** anxiety, depression, hukou, influence factor, STD clinic, urban migration

## Abstract

**Background:**

Sexually transmitted diseases (STDs) not only cause physical discomfort but also lead to significant psychological burdens, contributing to anxiety and depression among affected individuals. Understanding these psychological impacts is essential for effective STD management and prevention.

**Methods:**

This cross-sectional study surveyed 3,360 patients attending STD clinics at 10 randomly selected public medical institutions in Shanghai from September 2020 to December 2021. A structured questionnaire was used, covering demographics, sexual behavior, STD knowledge, and validated anxiety and depression scales (GAD-7 and PHQ-9). Multivariate logistic regression was employed to identify factors associated with anxiety and depression symptoms.

**Results:**

Anxiety and depression symptoms were reported by 10.8 and 13.9% of participants, respectively. Univariate analysis revealed higher prevalence among women, younger individuals, singles, those with poor STD knowledge, and individuals without local residency. Logistic regression identified age, marital status, local residence duration, registered residence, and STD knowledge as significant factors influencing anxiety, while gender, marital status, education, local residence duration, registered residence, and STD knowledge were key factors affecting depression. All ORs were reported with 95% confidence intervals.

**Conclusion:**

Addressing psychological health is crucial in STD management. This study identifies registered residence status, short-term urban residency, and the paradoxical association between higher education and increased depression risk as noteworthy findings in the Chinese urban context. Targeted interventions based on the identified risk factors may contribute to improved treatment compliance, potentially reduce incidence rates, and could be associated with enhanced patient outcomes and public health.

## Highlights

### What we already know

- Patients in STD clinics often experience higher levels of anxiety and depression due to disease-related stigma and psychosocial stressors.
- Psychological distress can impact patients’ ability to adhere to risk-reduction strategies and engage in preventive behaviors.
- Previous studies have explored the association between mental health and STDs, but there is limited research focusing on urban-specific factors influencing psychological distress in rapidly developing cities like Shanghai.

### What this article adds

- This study provides empirical data on the prevalence and influencing factors of anxiety and depression among STD clinic clients in Shanghai, highlighting the role of demographic and behavioral factors.
- It identifies knowledge gaps about STDs as a significant predictor of psychological distress, emphasizing the need for targeted educational interventions.
- Findings suggest that integrating mental health assessments into STD clinics can improve patient well-being and public health outcomes.

## 1 Introduction

Sexually transmitted diseases (STDs), including gonorrhea, syphilis, chlamydia, and trichomoniasis (1), are infections primarily transmitted through sexual contact, with over one million new cases reported globally each day according to WHO estimates.

The incidence and prevalence patterns of STDs are well aligned with the biopsychosocial model (2). Research has demonstrated that patients attending STD clinics are more vulnerable to depression and anxiety due to the unique challenges posed by sexually transmitted infections (3, 4). These psychological impacts are further compounded by self-imposed pressure and social stigma, all of which may profoundly impair their quality of life (5). Moreover, psychological conditions such as depression may impair patients’ ability to understand risk-reduction strategies and implement necessary behavioral changes (3, 6, 7). Therefore, it is imperative for healthcare practitioners in STD clinics to integrate mental health assessments into routine clinical practice (7). Employing psychological and cognitive-behavioral interventions may significantly improve patients’ awareness of STD-related risks, promote safer sexual practices, and may help reduce the risk of disease recurrence (8).

The relationship between mental health and STDs in rapidly urbanizing cities, such as Shanghai, is shaped by unique social and cultural factors. In particular, China’s hukou (household registration) system creates a structural stratification between local and non-local residents, which has been associated with differential access to healthcare and social support and may heighten psychological vulnerability among migrant populations (9). The hukou system determines access to public services, including healthcare, education, and social insurance, based on one’s place of registration rather than current residence; non-local residents, even those who have lived in Shanghai for years, may therefore face institutional barriers to care, potentially amplifying distress in the context of STD diagnosis. This migration-related dimension of psychological distress remains insufficiently understood. Moreover, the paradoxical association between higher educational attainment and elevated depression risk warrants further investigation, as it may reflect heightened stigma sensitivity, occupational pressure, or social image concerns among more educated individuals. However, there is a notable lack of research exploring how these urban-specific dynamics influence the mental health outcomes of STD clinic patients (9).

This study seeks to address these gaps by examining the prevalence of anxiety and depression among STD patients in Shanghai, thereby providing a foundation for the development of targeted psychological and educational interventions.

## 2 Materials and methods

### 2.1 Research population and procedures

A cross-sectional study was conducted among patients attending STD clinics from September 2020 to December 2021 in Shanghai, China. Using a multi-stage sampling method, we recruited participants from 10 randomly selected public medical institutions out of 62 in Shanghai, and an anonymous questionnaire survey was administered.

#### 2.1.1 First stage – sampling of medical institutions

The sampling frame consisted of all 62 public medical institutions in Shanghai with dedicated STD outpatient clinics. Ten institutions were randomly selected using computer-generated random numbers. This approach minimized selection bias and enhanced the representativeness of the sample across different administrative districts and hospital tiers in Shanghai.

#### 2.1.2 Second stage – recruitment of participants

To obtain a representative sample reflecting routine patient attendance, trained research staff consecutively approached all eligible patients in the waiting area during routine clinic hours at each selected study site.

The questionnaire included sociodemographic characteristics, STD-related healthcare behaviors, sexual behaviors, and measures assessed via the Generalized Anxiety Disorder Scale (GAD-7) and the Patient Health Questionnaire (PHQ-9), along with other relevant variables.

The inclusion criteria were as follows: (1) aged 18 years or older; (2) presenting to the STD clinics of the selected medical institutions; (3) no cognitive impairment; and (4) willing to participate and provide informed consent. Exclusion criteria included: (1) individuals aged younger than 18 years, (2) patients who declined participation; and (3) those unable to complete the survey owing to severe physical or psychiatric conditions.

Face-to-face interviews were conducted by trained investigators with patients in the waiting areas of the STD clinics. A total of 3,410 patients were surveyed, of which 3,360 completed valid questionnaires, yielding an overall response rate of 98.5% (3,360/3,410).

### 2.2 Data collection and measurement

#### 2.2.1 Sociodemographic information

Sociodemographic variables included sex, age, educational level, marital status, occupation, average monthly income, registered residence, and duration of residence in Shanghai. Registered residence (hukou) refers to an individual’s official household registration location as defined by China’s hukou system (9). In this system, public services such as healthcare subsidies, children’s schooling, and social insurance entitlements are tied to the place of registration rather than the place of actual residence. Individuals registered outside Shanghai (“non-Shanghai hukou”) may face structural disadvantages in accessing local health and social services even if they have resided in Shanghai for extended periods. Duration of local residence reflects the degree of social integration and is analysed separately from hukou status, as the two capture distinct dimensions of migration-related vulnerability.

#### 2.2.2 Sexual and healthcare-related behaviors

The collected data included whether participants engaged in sexual activity following a suspected STD infection, whether they had notified their sexual partners of their infection status, whether their partners had undergone STD testing, and whether the participants had received HIV testing in the past 3 months.

#### 2.2.3 Knowledge of STDs

A 15-item scale was adopted to assess participants’ knowledge of STDs, covering clinical diagnosis and treatment, preventive strategies, and consistent condom use. Content validity was established through expert review by five public health specialists with expertise in STD prevention and sexual health education. Items were evaluated for relevance, clarity, and comprehensiveness, and the scale was subsequently refined through a pilot study (n = 50) conducted prior to the main survey. Item-level feedback from the pilot was used to revise ambiguous phrasing. The internal consistency of the knowledge subscale in the current sample was acceptable (Cronbach’s *α* = 0.79). Participants who answered 9 or more items correctly (i.e., at or above the sample mean) were classified as having adequate STD knowledge. This mean-based cutoff approach is consistent with methods employed in prior domestic STD knowledge assessment studies (10).

#### 2.2.4 Anxiety and depression

GAD-7 and PHQ-9 were used to screen for symptoms of anxiety and depression. Participants reported how often they had experienced relevant symptoms over the preceding 2 weeks. Responses were categorized into four options: “not at all,” “several days,” “more than half the days,” and “almost every day,” with assigned scores of 0, 1, 2, and 3, respectively.

According to standard scoring criteria for the GAD-7, total scores ranged from 0 to 21, with categorizations as follows: no anxiety (0–4), mild anxiety (5–9), moderate anxiety (10–13), moderate to severe anxiety (14–18), and severe anxiety (19–21). A cutoff score of ≥ 10 points was defined as indicative of clinically relevant anxiety symptoms (11).

For the PHQ-9, total scores ranged from 0 to 27. The graded classifications included no depression (0–4), mild depression (5–9), moderate depression (10–14), moderately severe depression (15–19), and severe depression (20–27). A total score of ≥ 10 was classified as presenting depressive symptoms, consistent with widely validated diagnostic standards (12).

#### 2.2.5 Quality control

First, one-on-one interviews were administered in a quiet and private setting to ensure accurate and complete responses. Any missing or ambiguous items were clarified during the interview in accordance with ethical guidelines. Second, all investigators underwent professional training prior to the survey to ensure uniformity in survey administration, standardizing procedures and instructions for all participants.

The questionnaire was self-developed by the research team, revised based on consultations with public health experts, and validated through a pilot survey. The reliability of the questionnaire was satisfactory, with a Cronbach’s alpha value of 0.937. The validity index was 0.970, indicating good validity.

#### 2.2.6 Statistical analysis

A database was established using EpiData 3.0, and statistical analysis was performed with SPSS 27.0. Continuous variables were presented as mean ± standard deviation, while categorical variables were presented as frequencies and proportions. Between groups comparisons were conducted using the χ^2^ (chi-square) test. Binary logistic regression analysis was employed to identify factors influencing anxiety and depression in the target population. Odds ratios (ORs) with 95% confidence intervals (CIs) were reported for all regression coefficients. A *p* value of <0.05 was considered as statistically significant.

## 3 Results

### 3.1 Anxiety and depression levels

Among the 3,360 valid respondents, the mean GAD-7 score was 3.13 ± 4.85, and the mean PHQ-9 score was 3.93 ± 5.78. Based on the scoring criteria for the GAD-7 and PHQ-9 scales, 362 participants (10.8%) screened positive for anxiety symptoms (total score ≥10), and 467 participants (13.9%) screened positive for depressive symptoms (total score ≥10).

The average number of correct responses to the 15-item STD knowledge scale was 8.63 ± 3.92. Based on the mean-based accuracy threshold described above, respondents who answered 9 or more questions correctly were considered to have adequate STD knowledge. A total of 2,170 participants (64.6%) met this criterion.

### 3.2 Analysis of influencing factors

The factors influencing anxiety and depression among patients in STD clinics are presented in Tables 1, 2.

**Table 1:** Univariate analysis of anxiety symptoms among patients in STD clinics.

Variables	Total (*n* = 3,360)	Without anxiety symptoms (*n* = 2,998)	With anxiety symptoms (*n* = 362)	*χ* ^2^	*p*-value	OR	95% CI
Sex, *n* (%)							
Male	1,922 (57.2%)	1,736 (90.3%)	186 (9.7%)	5.616	0.018	1.302	1.046–1.619
Female	1,438 (42.8%)	1,262 (87.8%)	176 (12.2%)				
Age, *n* (%)							
≤30 years	820 (24.4%)	688 (83.9%)	132 (16.1%)	31.981	<0.001	0.519	0.412–0.653
>30 years	2,540 (75.6%)	2,310 (90.9%)	230 (9.1%)				
Education, *n* (%)							
Junior high school or lower	1,449 (43.1%)	1,311 (90.5%)	138 (9.5%)	4.141	0.042	1.261	1.008–1.578
High school or higher	1911 (56.9%)	1,687 (88.3%)	224 (11.7%)				
Marital status, *n* (%)							
Single	935 (27.8%)	789 (84.4%)	146 (15.6%)	31.585	<0.001	0.528	0.422–0.662
Married	2,425 (72.2%)	2,209 (91.1%)	216 (8.9%)				
Average monthly income, *n* (%)							
<6,000 (yuan)	2,358 (70.2%)	2,108 (89.4%)	250 (10.6%)	0.242	0.623	1.061	0.838–1.344
≥6,000 (yuan)	1,002 (29.8%)	890 (88.8%)	112 (11.2%)				
Registered residence, *n* (%)							
Shanghai	2,033 (60.5%)	1,867 (91.8%)	166 (8.2%)	36.437	<0.001	1.949	1.565–2.428
Non-Shanghai	1,327 (39.5%)	1,131 (85.2%)	196 (14.8%)				
Duration of residence in the local area, *n* (%)							
≤1 year	579 (17.2%)	486 (83.9%)	93 (16.1%)	20.352	<0.001	0.560	0.434–0.722
>1 year	2,781 (82.8%)	2,512 (90.3%)	269 (9.7%)				
Basic knowledge of STD, *n* (%)							
Unqualified	1,190 (35.4%)	1,032 (86.7%)	158 (13.3%)	12.833	<0.001	1.494	1.198–1.864
Qualified	2,170 (64.6%)	1970 (90.8%)	200 (9.2%)				
Notification of sexual partners, *n* (%)							
Yes	2,457 (73.1%)	2,214 (90.1%)	243 (9.9%)	7.427	0.006	1.383	1.094–1.747
No	903 (26.9%)	784 (86.6%)	119 (13.4%)				
Testing status of sexual partners, *n* (%)							
Yes	2,361 (70.3%)	2,133 (90.3%)	228 (9.7%)	10.304	0.001	1.449	1.154–1.819
No	999 (29.7%)	865 (86.6%)	134 (13.4%)				
HIV testing in the past 3 months, *n* (%)							
Yes	1,344 (40.0%)	1,215 (90.4%)	129 (9.6%)	3.220	0.073	1.231	0.981–1.545
No	2,016 (60.0%)	1,783 (88.4%)	233 (11.6%)				
Sexual activity after infection, *n* (%)							
Yes	983 (29.3%)	869 (88.4%)	114 (11.6%)	0.980	0.322	0.888	0.702–1.124
No	2,377 (70.7%)	2,129 (89.6%)	248 (10.4%)				

STD, sexually transmitted disease; HIV, human immunodeficiency virus; *n*, number.

**Table 2:** Univariate analysis of depression symptoms among patients in STD clinics.

Variables	Total (*n* = 3,360)	Without depressive symptoms (*n* = 2,893)	With depressive symptoms (*n* = 467)	*χ* ^2^	*p*-value	OR	95% CI
Sex, *n* (%)							
Male	1,922 (57.2%)	1,693 (88.1%)	229 (11.9%)	14.774	<0.001	0.682	0.561–0.830
Female	1,438 (42.8%)	1,200 (83.4%)	238 (16.6%)				
Age, *n* (%)							
≤30 years	820 (24.4%)	654 (79.8%)	166 (20.2%)	36.493	<0.001	0.530	0.430–0.652
>30 years	2,540 (75.6%)	2,239 (88.1%)	301 (11.9%)				
Education, *n* (%)							
Junior high school or lower	1,449 (43.1%)	1,274 (87.9%)	175 (12.1%)	7.064	0.008	1.313	1.074–1.606
High school or higher	1,911 (56.9%)	1,619 (84.7%)	292 (15.3%)				
Marital status, *n* (%)							
Single	935 (27.8%)	749 (80.1%)	186 (19.9%)	38.897	<0.001	0.528	0.431–0.647
Married	2,425 (72.2%)	2,144 (88.4%)	281 (11.6%)				
Average monthly income, *n* (%)							
<6,000 (yuan)	2,358 (70.2%)	2,042 (86.6%)	316 (13.4%)	1.636	0.204	1.143	0.930–1.414
≥6,000 (yuan)	1,002 (29.8%)	851 (84.9%)	151 (15.1%)				
Registered residence, *n* (%)							
Shanghai	2,033 (60.5%)	1,820 (89.5%)	213 (10.5%)	50.361	<0.001	2.023	1.661–2.463
Non-Shanghai	1,327 (39.5%)	1,073 (80.9%)	254 (19.1%)				
Duration of residence in the local area, *n* (%)							
≤1 year	579 (17.2%)	461 (79.6%)	118 (20.4%)	24.555	<0.001	0.561	0.445–0.706
>1 year	2,781 (82.8%)	2,432 (87.5%)	349 (12.5%)				
Basic knowledge of STD, *n* (%)							
Unqualified	1,190 (35.4%)	988 (83.0%)	202 (17.0%)	14.568	<0.001	0.680	0.558–0.830
Qualified	2,170 (64.6%)	1905 (87.8%)	265 (12.2%)				
Notification of sexual partners, *n* (%)							
Yes	2,457 (73.1%)	2,126 (86.5%)	331 (13.5%)	1.394	0.238	1.139	0.918–1.414
No	903 (26.9%)	767 (84.9%)	136 (15.1%)				
Testing status of sexual partners, *n* (%)							
Yes	2,361 (70.3%)	2,054 (87.0%)	307 (13.0%)	5.325	0.021	1.276	1.037–1.570
No	999 (29.7%)	839 (84.0%)	160 (16.0%)				
HIV testing in the past 3 months, *n* (%)							
Yes	1,344 (40.0%)	1,177 (87.6%)	167 (12.4%)	4.062	0.044	1.232	1.006–1.510
No	2,016 (60.0%)	1,716 (85.1%)	300 (14.9%)				
Sexual activity after infection, *n* (%)							
Yes	983 (29.3%)	845 (86.0%)	138 (14.0%)	0.023	0.88	0.984	0.794–1.219
No	2,377 (70.7%)	2,048 (86.2%)	329 (13.8%)				

STD, sexually transmitted disease; HIV, human immunodeficiency virus; *n*, number.

As shown in Table 1, univariate *χ*^2^ analyses demonstrated that female sex, younger age (≤30 years), higher educational level, single marital status, non-Shanghai registered residence, shorter local residence duration (≤1 year), unqualified STD knowledge, failure to notify sexual partners, and lack of partner STD testing were all significantly associated with a higher prevalence of anxiety symptoms (all *p* < 0.05). In contrast, average monthly income, recent HIV testing, and sexual activity after infection showed no significant association with anxiety (all *p* > 0.05).

As shown in Table 2, univariate analyses demonstrated that female sex, younger age (≤30 years), higher educational level, single marital status, non-Shanghai registered residence, shorter local residence duration (≤1 year), unqualified STD knowledge, lack of partner STD testing, and no recent HIV testing were all significantly associated with a higher prevalence of depressive symptoms (all *p* < 0.05). In contrast, average monthly income, sexual partner notification, and sexual activity after infection showed no significant association with depressive symptoms (all *p* > 0.05).

### 3.3 Factors influencing anxiety and depression among patients in STD clinics

Binary logistic regression was used to analyze the factors influencing anxiety and depression among patients in sexually transmitted disease clinics. The Hosmer–Lemeshow test showed *p* values greater than 0.05, indicating adequate fit of the logistic regression models.

**Figure 1:**
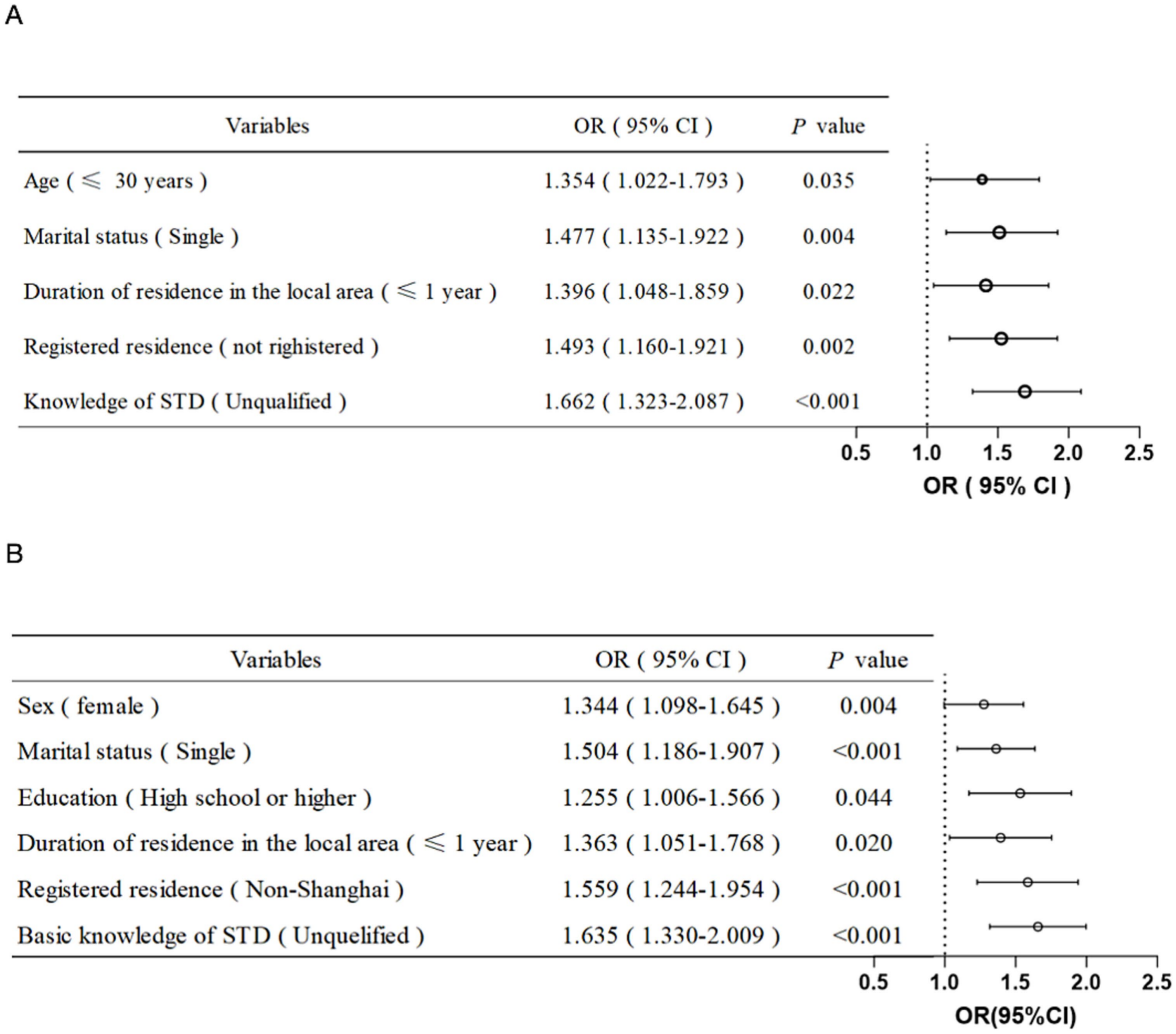
Logistic regression analysis of anxiety and depression related risk factors. **(A)** Forest plot of multivariate logistic regression for factors associated with anxiety symptoms. **(B)** Forest plot of multivariate logistic regression for factors associated with depression symptoms.

Binary logistic regression analysis revealed five independent risk factors for anxiety symptoms: younger age (≤30 years, OR = 1.354, 95% CI: 1.022–1.793, *p* = 0.035), single marital status (OR = 1.477, 95% CI: 1.135–1.922, *p* = 0.004), shorter local residence duration (≤1 year, OR = 1.396, 95% CI: 1.048–1.859, *p* = 0.022), non-Shanghai registered residence (OR = 1.493, 95% CI: 1.160–1.921, *p* = 0.002), and poor STD knowledge (OR = 1.662, 95% CI: 1.323–2.087, *p* < 0.001) (Figure 1A).

Binary logistic regression analysis revealed six independent risk factors for depressive symptoms: female (OR = 1.344, 95% CI: 1.098–1.645, *p* = 0.004), single marital status (OR = 1.504, 95% CI: 1.186–1.907, *p* < 0.001), higher educational level (OR = 1.255, 95% CI: 1.006–1.566, *p* = 0.044), shorter local residence duration (≤1 year, OR = 1.363, 95% CI: 1.051–1.768, *p* = 0.020), non-Shanghai registered residence (OR = 1.559, 95% CI: 1.244–1.954, *p* < 0.001), and poor STD knowledge (OR = 1.635, 95% CI: 1.330–2.009, *p* < 0.001). All factors were positively associated with increased odds of depressive symptoms (Figure 1B).

## 4 Discussion

This study revealed that 10.8% of respondents exhibited symptoms of anxiety and 13.9% exhibited symptoms of depression. While some identified risk factors, such as younger age, female sex, and single marital status, are consistent with patterns observed in international STD mental health literature, several findings offer insights that are particularly relevant to the Chinese urban context. Specifically, hukou (registered residence) status and short-term local residency emerged as independent predictors of both anxiety and depression, and higher educational attainment was paradoxically associated with elevated depression risk.

### 4.1 Anxiety and depression of patients in STD clinics

The prevalence rates of anxiety and depression observed in this study are lower than those reported by Erbelding et al. (6), who found substantially higher rates of depressive symptoms in a US-based STD clinic population. Cultural, social, and demographic differences might explain these discrepancies. Prevalence rates in our study are aligned with Wilson et al. (7), who emphasized the critical role of STD clinics in identifying and addressing mental health conditions. Some scholars suggest that psychological care can significantly improve the mental well-being, immunity, and overall quality of life of STD patients (13). Effective psychological assessment in STD clinics is essential for accurate diagnosis, treatment, and prevention (14). Medical staff should build trust through non-judgmental communication, active listening, and empathetic engagement, while also offering psychological support to patients’ families to create a supportive care environment. Our findings are broadly consistent with prior Chinese domestic research: Liu et al. (10) reported similarly elevated rates of psychological distress among 263 STD patients in a Chinese urban clinic, suggesting that the mental health burden in this population is not unique to Western settings but may be shaped by distinct local dynamics, including the structural pressures of the hukou system.

Consistent with previous studies, our findings underscore that social stigma, poor disease knowledge, and inadequate social support contribute to psychological distress (15–17). Notably, we observed that higher educational attainment was independently associated with increased depression risk, a finding that warrants careful interpretation.

We propose three possible explanatory pathways for this counterintuitive finding. First, social image sensitivity: highly educated individuals may be more attuned to the social implications of STD diagnosis, perceiving it as a greater threat to their professional reputation and family relationships, thereby amplifying perceived stigma and depressive burden (18). Second, relative deprivation: among migrant populations in Shanghai, some highly educated individuals may occupy positions below their expected socioeconomic status (“over-qualified underemployment”), generating a sense of relative deprivation that interacts with STD-related stress to increase depression risk. Third, reporting willingness: individuals with greater health literacy may be more willing to acknowledge and report depressive symptoms, potentially inflating observed rates in higher-education subgroups. As the current cross-sectional design cannot disentangle these mechanisms, future research incorporating measures of occupational status, stigma sensitivity, and health literacy is warranted to test these hypotheses.

Younger and single patients demonstrated higher incidences of anxiety and depression, likely stemming from concerns about future relationships, fears of transmitting infections to partners, fears of transmitting infections to future generations, and the resulting emotional burden. Female patients were also found to have a higher susceptibility to depression, consistent with patterns reported in broader mental health literature and prior STD-related studies (6).

Univariate analysis indicates that anxiety symptoms are more prevalent among individuals who did not inform their sexual partners or whose partners did not undergo testing, which may reflect social stigma, concerns about public opinion, and familial dynamics. Additionally, patients who have not undergone HIV testing within the past 3 months show significantly higher depression rates, suggesting an internal conflict post-infection that warrants focused psychological support (16, 19).

### 4.2 Mechanistic insights

*Social support and residence status*: a particularly noteworthy finding is the independent association of both non-Shanghai registered residence and short-term local residency with elevated anxiety and depression. In the context of China’s hukou system, non-local residents may face limited access to subsidised healthcare, reduced social support networks, and heightened concerns about treatment costs and employment disruption (20). Short-term residency may additionally reflect lower social integration, leaving individuals more isolated at the time of STD diagnosis. These findings are consistent with Arkell et al. (20) and underscore the importance of considering structural determinants of health in STD mental health research.

*Disease knowledge and psychological distress*: insufficient knowledge about STDs is another key factor contributing to depression among patients. Poor understanding of the disease often leads to uncertainty and fear, intensifying psychological burden and emotional distress (21).

*Behavioral consequences of psychological distress*: anxiety and depression symptoms may discourage patients from notifying sexual partners, adhering to treatment, or undergoing regular testing, thereby increasing the risk of disease transmission.

### 4.3 Practical implications

We propose a two-stage clinic-based strategy to address the psychological needs of STD patients. In the first stage, routine screening should be integrated into initial consultations: the PHQ-2 and GAD-2 can serve as rapid first-line tools, with positive screens triggering brief psychological education delivered by trained nursing staff and referral to counselling services (with privacy protections in place) (22). In the second stage, STD clinic physicians should receive training in low-intensity psychological support techniques, including normalising emotional responses to diagnosis and providing anti-stigma messaging. Formal referral pathways to psychiatric services should be established for patients requiring pharmacological intervention. Digital platforms, including instant messaging and app-based follow-up, can supplement clinic contact, improve partner notification rates, and enhance treatment adherence. For migrant patients with non-local hukou status, linkage to community-based social support services may help address the structural vulnerabilities identified in this study.

### 4.4 Limitations

This study has several important limitations that should be considered when interpreting the findings. First, the cross-sectional design precludes causal inference: it is not possible to determine whether psychological distress precedes or follows STD diagnosis and the associated social stressors (reverse causality cannot be excluded). Second, all data were obtained through self-report, introducing the potential for recall bias and reporting bias. In particular, the dual stigma associated with STDs and mental health difficulties in Chinese society may have led some participants to underreport psychological symptoms or minimise descriptions of sexual risk behaviours, potentially underestimating the true prevalence of distress. Third, several clinically important variables were not collected, including STD diagnosis type, primary versus recurrent infection, symptom severity, and treatment stage. These factors may independently or interactively influence psychological outcomes, and their absence represents a notable limitation of the present analysis. Fourth, although the STD knowledge scale was content-validated by public health experts and pilot-tested, it has not undergone formal psychometric validation including confirmatory factor analysis or external criterion validation; the mean-based scoring cutoff, while pragmatic, lacks a standardised evidence base. Fifth, the study was conducted in public STD clinics in Shanghai and may not be representative of patients in private clinics, rural settings, or other Chinese cities. Future research should adopt longitudinal and multicenter designs, incorporate validated measurement instruments, and collect comprehensive clinical data to confirm and extend these findings.

## 5 Conclusion

The psychological state of patients in STD clinics may have a meaningful impact on clinical treatment outcomes and disease prevention. This study identified several risk factors for anxiety and depression among STD clinic patients in Shanghai, with hukou (registered residence) status and short-term local residency emerging as contextually relevant predictors. The paradoxical association between higher educational attainment and increased depression risk also warrants further investigation. Medical staff should integrate routine psychological screening into STD clinic consultations, identify high-risk populations using validated tools, and implement targeted health education and psychological interventions. Such approaches may contribute to improved knowledge, higher partner treatment rates, better treatment adherence, and potentially lower recurrence rates of sexually transmitted diseases. The findings of this study may provide a useful basis for healthcare providers and policymakers seeking to design effective health education and psychological intervention programmes for this population.

## Data Availability

The original contributions presented in the study are included in the article/supplementary material, further inquiries can be directed to the corresponding author.
